# Advances in the Early Detection of Hepatobiliary Cancers

**DOI:** 10.3390/cancers15153880

**Published:** 2023-07-30

**Authors:** Hasan Çağrı Yıldırım, Gozde Kavgaci, Elvin Chalabiyev, Omer Dizdar

**Affiliations:** Department of Medical Oncology, Faculty of Medicine, Hacettepe University, 06230 Ankara, Turkey; hasan-cagri@windowslive.com (H.Ç.Y.); gozdekavgaci@hacettepe.edu.tr (G.K.); elvin.chelebi@gmail.com (E.C.)

**Keywords:** biomarkers, early detection, hepatocellular cancer, biliary tract cancer

## Abstract

**Simple Summary:**

Hepatocellular cancer and biliary tract cancers are associated with poor prognosis, particularly in advanced stages. However, early diagnosis offers the potential for curative treatment. In this review, our objective was to compile the standard diagnostic tests recommended for early detection in individuals with specific risk factors, as well as to review the recent advancements in this field.

**Abstract:**

Hepatocellular cancer (HCC) and biliary tract cancers (BTCs) have poor survival rates and a low likelihood of a cure, especially in advanced-stage disease. Early diagnosis is crucial and can significantly improve survival rates through curative treatment approaches. Current guidelines recommend abdominal ultrasonography (USG) and alpha-fetoprotein (AFP) monitoring for HCC screening in high-risk groups, and abdominal USG, magnetic resonance imaging (MRI), and magnetic resonance cholangiopancreatography (MRCP) monitoring for biliary tract cancer. However, despite this screening strategy, many high-risk individuals still develop advanced-stage HCC and BTC. Blood-based biomarkers are being developed for use in HCC or BTC high-risk groups. Studies on AFP, AFP-L3, des-gamma-carboxy prothrombin, glypican-3 (GPC3), osteopontin (OPN), midkine (MK), neopterin, squamous cell carcinoma antigen (SCCA), Mac-2-binding protein (M2BP), cyclic guanosine monophosphate (cGMP), and interleukin-6 biomarkers for HCC screening have shown promising results when evaluated individually or in combination. In the case of BTCs, the potential applications of circulating tumor DNA, circulating microRNA, and circulating tumor cells in diagnosis are also promising. These biomarkers have shown potential in detecting BTCs in early stages, which can significantly improve patient outcomes. Additionally, these biomarkers hold promise for monitoring disease progression and evaluating response to therapy in BTC patients. However, further research is necessary to fully understand the clinical utility of these biomarkers in the diagnosis and management of HCC and BTCs.

## 1. Hepatocellular Cancer

Hepatocellular carcinoma (HCC) is the third leading cause of cancer-related deaths globally [1]. In 2020, HCC caused an estimated 830,180 deaths worldwide. HCC incidence varies significantly based on geographic location, with Asia being the most affected region, accounting for 72% of cases. Other regions, such as Europe, Africa, North America, and Latin America, have a lower incidence [2]. HCC is particularly prevalent in sub-Saharan Africa and Southeast Asia [3]. Unfortunately, the disease is often diagnosed at advanced stages, with only 43% of patients diagnosed in the early stages, and the 5-year survival rate is only 35% [4]. Patients with advanced disease survive a few months, whereas those with early-stage disease have a five-year survival rate of 60–70% with appropriate treatment. Therefore, the screening and prevention of HCC are critical.

### 1.1. Etiology

Several risk factors for HCC have been identified, whereby most of which involve liver injury leading to cirrhosis, a condition present in 90% of HCC patients [5]. Up to one third of patients with cirrhosis will develop HCC during their lifetime, with an annual incidence rate estimated to be between 1% and 8% [6]. Chronic hepatitis B virus (HBV) infection is responsible for more than 50% of HCC cases worldwide, especially in Asian countries [5]. Vaccination against HBV is an effective measure to provide primary immunity [7]. Chronic HCV infection increases the risk of HCC by an average of 17 times [4]. HCV infection is often asymptomatic, and early detection through screening can significantly reduce the incidence of HCV-related cirrhosis and the risk of HCC [4]. Other significant causes of HCC include metabolic disorders such as non-alcoholic fatty liver disease (NAFLD) and non-alcoholic steatohepatitis (NASH), alcoholic liver disease, obesity, exposure to toxic substances such as aflatoxin B1, vinyl chloride monomers, and organic solvents, hemochromatosis, alpha-1 antitrypsin deficiency, acute intermittent porphyria, and hereditary tyrosinemia [8].

The incidence of NAFLD and NASH is rapidly increasing worldwide, and they can lead to serious consequences if left untreated. NAFLD/NASH patients are at a seven-fold increased risk of developing HCC compared to healthy individuals [9]. The mortality rate also increases with fibrosis levels, ranging from 0.1% for F0 to 34.6% for F4. Therefore, the early diagnosis and management of NAFLD/NASH patients are crucial to prevent disease progression and reduce associated risks. The definition of NAFLD has been expanded to include metabolic dysfunction, resulting in the use of the term “metabolic-associated fatty liver disease” (MAFLD) [10]. For MAFLD patients with a liver stiffness value greater than 15 kPa, biannual ultrasonography (USG) and alpha-fetoprotein (AFP) measurements are recommended for HCC screening [10].

Alcoholic cirrhosis is another liver disease caused by excessive alcohol consumption over an extended period, leading to liver cell death and scar tissue formation [11]. The risk of HCC development in patients with alcoholic cirrhosis is influenced by various factors, including the amount and duration of alcohol consumption, gender, advanced age, obesity, type 2 diabetes, the PNPLA3 variant, and gut microbial dysbiosis [11]. Regular monitoring and appropriate measures to reduce the risk of HCC development are crucial for effectively managing alcoholic cirrhosis.

HCC surveillance is generally recommended for patients who are at high risk of developing liver cancer. This includes patients with cirrhosis, particularly those with Child–Pugh stages A and B, and those with cirrhosis awaiting liver transplantation with Child–Pugh stage C [12]. Patients with chronic hepatitis B virus (HBV) infection in the absence of cirrhosis are also at high risk and should undergo surveillance. Additionally, non-cirrhotic patients with advanced fibrosis (F3), regardless of etiology, may also be considered for surveillance based on individual risk assessment [13,14].

### 1.2. Imaging

Abdominal ultrasound is the current standard for HCC surveillance, which should be performed every six months by experienced personnel in all high-risk populations. In a population-based cluster-randomized study from China, surveillance with AFP and USG reduced HCC-related mortality compared with no surveillance in patients with chronic HBV infection with or without cirrhosis [15]. USG is widely available, non-invasive, and has a relatively lower cost, making it an optimal surveillance method. However, it has limitations, such as dependence on the expertise of the operator and technical disadvantages like low sensitivity in a cirrhotic background and in obese patients. CT or MRI are more sensitive but are associated with high cost, administration of contrast, radiation exposure (for CT), and false positive results, which preclude their use as a primary surveillance strategy [16]. CT or MRI can be used if visualization via USG is poor, such as in obese patients. A meta-analysis of cohort studies examining hepatic ultrasonography for the identification of early-stage HCC found that the pooled sensitivity was 45%, increasing to 63% when alpha-fetoprotein (AFP) was included [17].

Contrast-enhanced ultrasound (CEUS) involves the injection of a contrast agent and the use of ultrasound to produce real-time images of the liver vasculature. CEUS significantly improves the diagnostic accuracy of ultrasound in the detection and characterization of focal liver lesions [18]. Specifically, the CEUS Liver Imaging Reporting and Data System (LI-RADS) provides standardized terminology, interpretation, and reporting for the diagnosis of HCC. In addition to its diagnostic accuracy, CEUS has other advantages over CT and MRI. CEUS does not involve ionizing radiation and is less expensive and more widely available than CT in many healthcare settings [19]. On the other hand, similar limitations apply for CEUS and conventional USG, including operator dependency and limited sensitivity in obese patients or deeply localized lesions in the liver. CEUS can be used to further characterize suspicious nodules, but currently it is not recommended to be used for surveillance and routine staging in EASL, AASLD, or NCCN guidelines.

### 1.3. Serum Markers

AFP is the most widely used biomarker in HCC. AFP may increase the sensitivity of USG when used in combination in patients with cirrhosis. However, the specificity of AFP is low, as it can also be increased in flares of HBV/HCV infection, cirrhosis, or other underlying liver diseases. Another limitation is that AFP usually increases in later stages of HCC, precluding its use in early diagnosis. A systematic review showed that the sensitivity of AFP was 41% to 65%, and the specificity was 80% to 94%, with a cutoff value of >20 µg/L [20]. AFP levels may be within normal limits in up to 30–40% of patients with HCC. Other biomarkers such as lens culinaris agglutinin-reactive AFP (AFP-L3) and des-gamma-carboxy prothrombin (DCP) have been studied as potential diagnostic tools, but their clinical utility in HCC surveillance remains limited [14,16,21]. AFP-L3 is a glycoform of AFP and the AFP-L3 to AFP ratio has higher sensitivity and specificity to detect HCC [22]. DCP production does not increase in chronic liver disease; hence, it was suggested to be a more specific biomarker compared with AFP. However, a recent study showed that adding DCP did not further improve the sensitivity of AFP and AFP-L3 combination in patients with cirrhosis and/or chronic hepatitis B [23].

Combining tumor markers with ultrasound may improve HCC detection accuracy. A combination of ultrasound and DCP had higher sensitivity and specificity than ultrasound alone for detecting early HCC, according to a systematic review and meta-analysis [24,25]. Other studies have found that using a panel of three different HCC markers (AFP, DCP, and OPN) together had a higher sensitivity rate than using each marker alone [26,27,28]. The study involved 540 patients with HCC and 279 control individuals without HCC. The sensitivity of AFP alone was 64.6%, while the sensitivity of the three-marker panel was 80.0%. Moreover, the specificity of the three-marker panel was 91.4%, which was higher than the specificity of AFP alone (85.3%). The authors concluded that combining these three biomarkers could improve the accuracy of HCC diagnosis and reduce false negative results. In the study of Jorge et al., GP73 was better than AFP for the diagnosis of early HCC [29]. Several other biomarkers are suggested for use in the early diagnosis and prognostication of HCC, including osteopontin (OPN), midkine (MK), neopterin, squamous cell carcinoma antigen (SCCA), Mac-2-binding protein (M2BP), cyclic guanosine monophosphate (cGMP), and interleukin-6 (IL-6) [30,31,32,33,34]. Some of these biomarkers are extensively used in Japan [35,36]. Currently, these biomarkers are not recommended to be used in the surveillance of high-risk patients for the early diagnosis of HCC. The combination of blood-based biomarkers might have a role in the early detection of HCC in future.

Because of its vast blood supply, the liver is a substantial producer of cell-free DNA (cfDNA) [37]. A pan-cancer early detection study showed that HCC has the highest concentration of cfDNA shedding into the circulation compared to other malignancies, suggesting plasma cfDNA as a suitable option for early HCC diagnosis [38]. However, it is crucial for a cfDNA-based test to accurately differentiate between stages of chronic liver disease, such as liver cirrhosis, HBV/HCV infection, and HCC, as patients with liver illnesses are the target group for current screening guidelines. Therefore, findings from studies that primarily focused on HCC and healthy controls may not be directly applicable to the real-world screening population.

Recent studies have demonstrated the feasibility of using methylation markers to distinguish between HCC and non-HCC liver diseases [39]. Hypermethylation of specific genes has been identified as an early event in hepatocellular carcinoma (HCC) development. A six-marker MDM (methylation differential marker) panel exhibited the ability to distinguish HCC from cirrhosis and healthy controls, achieving sensitivity of 95% and specificity of 92%. Moreover, the panel successfully detected 91.3% of early-stage HCC cases (Barcelona Clinic Liver Cancer stages 0/A), accurately identifying 42 out of 46 patients [40]. Another study demonstrated comparable performance, wherein the methylation model exhibited sensitivity of 83.6% and specificity of 96% in a validation cohort comprising 67 HCC patients, along with 353 patients with liver cirrhosis and healthy controls [41].

Multi-omics approaches have also been evaluated to detect the presence of HCC. These approaches entail the integration of mutations in genes such as TP53, AXIN1, CTNNB1, and the promoter region of TERT, in addition to the HBV sequence and protein markers like AFP and DCP. Furthermore, clinical covariates such as age and sex are also taken into account [42]. The method was initially trained using a case–control design and then validated using a prospective cohort of asymptomatic individuals who tested positive for the HBV surface antigen [43]. The integrated multi-analytical approach demonstrated sensitivity of 85% and specificity of 93% in the training cohort. In the prospective cohort, 4 out of 24 individuals who tested positive were subsequently diagnosed with HCC through CT scans, MRI, or pathological review within 6–8 months after the baseline test. These tumors were all less than 3 cm in diameter when diagnosed. However, it is important to consider the limited time window of follow-up when evaluating the final specificity and sensitivity of the approach. Large-scale prospective studies of asymptomatic individuals at high risk for HCC are still lacking, despite recent progress. The ability of these new cfDNA markers to accurately identify patients with early-stage HCC who may benefit from liver resection or transplantation must be evaluated further. False positive cfDNA test results in patients with cirrhosis or HBV/HCV infection are also a concern, given that these liver diseases share similar genetic or epigenetic alterations to HCC [44,45]. Recent studies on diagnostic markers are shown in Table 1.

MicroRNAs are non-coding RNAs and have a function in the post-transcriptional regulation of gene expression. Recent studies have shown that serum miRNAs have high diagnostic value for HCC, and the diagnostic accuracy improves further when a combination of multiple miRNAs is used along with AFP. Pooled sensitivity was 0.874 (95% CI, 0.839–0.903), specificity was 0.875 (95% CI, 0.835–0.906), and AUC-SROC was 0.94 [46]. Several aberrant miRNA profiles, including mir-21, mir-192, mir-155, mir-224, mir-665, and mir-718, have been identified in patients with HCC, with varying diagnostic yields in different studies [47,48]. However, an optimal panel has not been defined yet. Long non-coding RNAs have also gained interest in the early diagnosis of HCC. Chen et al. showed, in a meta-analysis, that the pooled sensitivity and specificity were 0.87 (0.838–0.897) and 0.829 (0.794–0.86), and the diagnostic accuracy of lncRNAs was estimated in HCC with an AUC of 0.915 [49]. A panel of lncRNA alone or in combination with other biomarkers may serve as an important early diagnostic tool.

**Figure 1:**
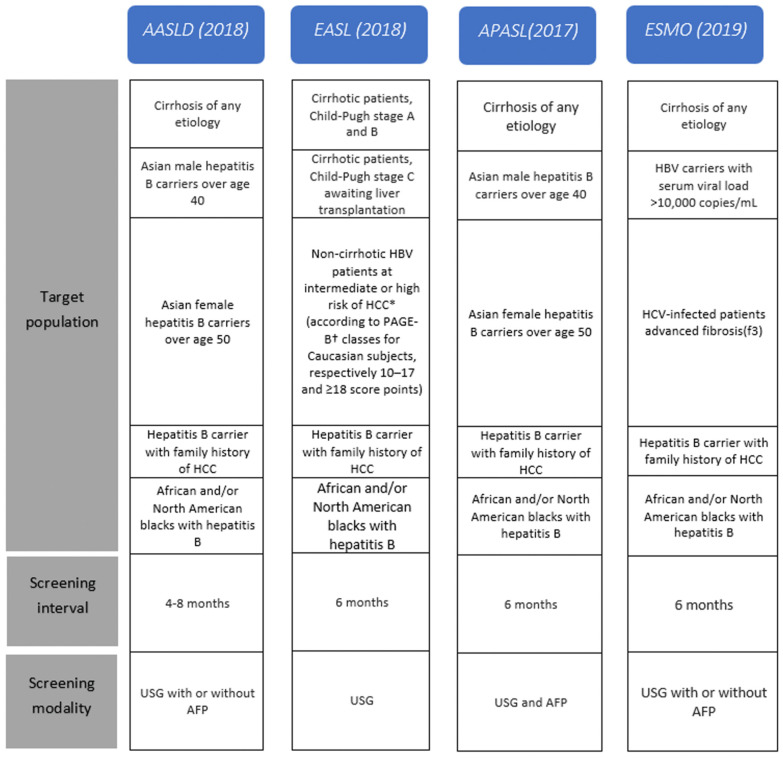
Hepatocellular cancer screening protocols of international guidelines. * Hepatocellular cancer.

**Figure 2:**
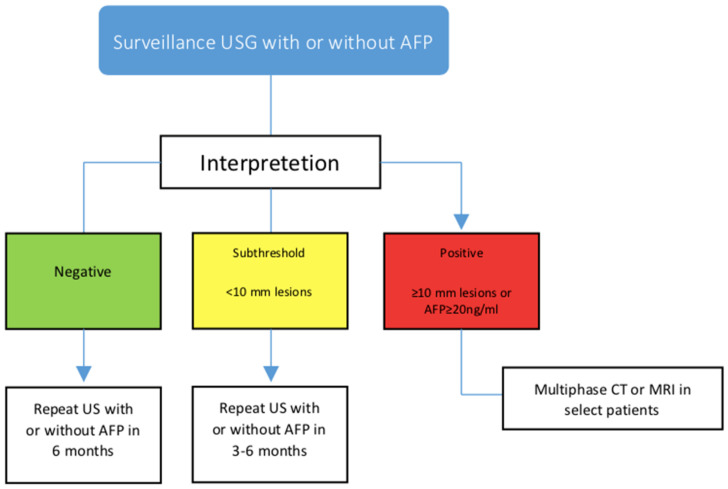
Follow-up protocol according to ultrasonography and AFP results.

HCC surveillance recommendations are based on an evaluation of their individual risk factors and a cost–benefit analysis of different surveillance strategies [50]. The individual risk factors that are considered include chronic hepatitis B or C infection and family history of HCC, obesity, and diabetes. The cost–benefit analysis takes into account the cost of screening tests, such as abdominal ultrasonography, and the potential benefits of detecting HCC early, when it may be more treatable. In general, surveillance intervals range from every 3–6 months, depending on the patient’s risk factors and the specific guidelines being followed [51]. However, it is important to note that these recommendations may vary among different organizations and countries (Hepatocellular cancer screening protocols of international guidelines are shown in Figure 1 and according to follow-up protocol to ultrasonography and AFP results are shown in Figure 2). Therefore, it is important for healthcare providers to personalize surveillance strategies based on each patient’s individual risk factors. For non-cirrhotic patients with HBV, the decision to recommend surveillance for HCC should be based on an evaluation of the patient’s individual risk factors and a cost–benefit analysis of different surveillance strategies. Expert opinion suggests that surveillance may be appropriate if the incidence of HCC is at least 0.2% per year [52]. However, a personalized screening approach may be more cost-effective, as it balances the need for early detection with the need to minimize unnecessary testing and interventions. 

**Table 1:** Efficacy of markers that can be used in the early diagnosis of hepatocellular cancer.

Marker	Method	Sensitivity	Specificity	Comment	Reference
AFP-L3	ELISA	36–96%	89–94%	AFP-L3 seems to be more reliable and have better prognostic value than total AFP in patients with HCC.	[53,54]
DCP	ELISAor İHC on tissue	28–89%	87–96%	DCP more specific for HCC, unaffected by other liver diseases (e.g., chronic hepatitis C), and is correlated with the HCC stage and survival.	[27,28]
GP73	Serumimmunoblot and densitometric analysis	69%	75%	Serum levels of GP73 are higher in patients with HCC than in those without the disease. GP73 was superior to AFP for the detection of early HCC.	[29]
GPC3	Western blotting and ELISA	50–72%	40–53%	Patients with HCC have substantially elevated serum GPC3 levels compared to healthy volunteers and patients with noncancerous liver diseases.	[55]
CTDNA	NGS	100%, 85%	94%, 93%	Combining the detection of cfDNA alterations and protein markers is a viable method for identifying HCC at an early stage.	[42,43]
Methylation markers	NGS	95%, 84%	92%, 96%	Plasma testing has been shown to accurately detect HCC.	[40,41]
miRNA	NGS	85–90%	87%, 80%	A combination of conventional molecular targeting agents and miRNA-based interventions for HCC could enhance transgene expression and gene transfer in primary and metastatic HCC.	[46,48]
lncRNA	NGS	87%	82%	lncRNAs are promising markers for diagnosis and prognosis, and may predict response to radiotherapy and systemic treatment.	[49]

## 2. Biliary Tract Cancer

Biliary tract cancers consist of gallbladder cancer and cholangiocarcinomas (intrahepatic, perihilar, and distal). Biliary tract cancers have a high mortality, so early detection is indeed the key to receiving treatment and reducing death rates.

### 2.1. Gallbladder Cancer

#### 2.1.1. Etiology

Gallbladder cancer is the most prevalent bile system malignancy and has a dismal prognosis. Often, it is diagnosed at advanced stages, often after a cholecystectomy for gallstones [56].

The dismal prognosis is due to both the lack of symptoms in the disease’s early stages and the aggressive nature of the disease. Most gallbladder cancers are of epithelial origin with cholangiocyte differentiation, with adenocarcinomas being the most common histological type [57]. The optimal circumstance is an early diagnosis, where cholecystectomy is the cure. Screening all patients for gallbladder cancer is not practical nor efficient, so it is important to consider the known risk factors and determine the population to screen [58].

Gallstones, gallbladder polyps, porcelain gallbladder, pancreaticobiliary maljunctions, primary sclerosing cholangitis, Salmonella enterica serovar Typhi infection, obesity, diabetes, metabolic syndrome, tobacco use, chemical exposure (mustard oil, aflatoxin, organochlorines), and heavy metals have been identified as risk factors for gallbladder cancer [59,60,61,62,63,64,65,66,67].

Gallstones with the presence of chronic inflammation are a well-established risk factor for gallbladder cancer [68,69]. Despite the fact that gallstones are present in 70–90% of patients with gallbladder cancer [70,71,72], the incidence of gallbladder cancer in patients with cholelithiasis is only 0.5–3% [73,74]. Surveillance with abdominal ultrasonography may be considered for patients with large stones, especially 3 cm or larger [75], and symptomatic gallbladder disease, for the possibility of coexisting cancer. Currently, no screening tests or recommended imaging modalities exist for use in patients with gallstones.

Porcelain gallbladder is a relatively uncommon but clinically important condition due to its relation with gallbladder cancer. It is characterized by intramural calcification of the gallbladder wall, associated with cholelithiasis, and chronic inflammation results in scarring, hyalinization, and calcification [76]. Recent studies have shown that the risk of gallbladder cancer in porcelain gallbladder is lower than anticipated and is between 6% and 15% [77,78]. Asymptomatic patients with a porcelain gallbladder may undergo prophylactic cholecystectomy according to the European Association for the Study of the Liver, but the evidence supporting prophylactic cholecystectomy is of low quality because a causal relationship was not established in all series [77,79].

Gallbladder polyps are usually found incidentally upon abdominal ultrasonography and occur in 1–7% of the general population [79,80,81,82,83]. Most gallbladder polyps are non-neoplastic, benign polyps with no malignant potential. The prevalence of adenomas with malignancy potential in people with gallbladder polyps is under 5% [81,84]. The size of the polyp is the major risk factor for malignancy since adenomatous polyps of 10 mm and larger have up to a 50% risk of malignancy [81,82,85,86,87,88]. A distinction between adenomas and non-adenomas is usually made after surgery; the decision to conduct surveillance of gallbladder polyps is critical if surgery is not performed [89]. The guidelines recommend that the primary investigation of polypoid lesions of the gallbladder should be with abdominal ultrasound and do not recommend the routine use of other imaging modalities [90]. Contrast-enhanced ultrasound and endoscopic ultrasound may be helpful. Cholecystectomy is recommended for people who have polyps that are 10 mm or larger or who have polyps and symptoms that could be caused by the gallbladder. For polyps 5 mm or less, a follow-up ultrasound of the gallbladder is recommended at 6 months, 1 year, and 2 years for patients with risk factors for malignancy, and no follow-up is recommended for patients without any risk factors. During follow-up, if the polyp grows to 10 mm, cholecystectomy is recommended, and if the polyp grows by 2 mm or more, cholecystectomy or the continuation of monitoring should be considered with patient risk factors. Cholecystectomy should be considered in patients with polyps between 6 and 10 mm and in cases of growing polyps. Endosonography may be helpful to differentiate gallbladder polyps of 6–10 mm in size from those that cause suspicions of gallbladder cancer. For polyps between 6 and 10 mm, follow-up ultrasound or endosonography of the gallbladder are recommended every 3 to 6 months initially and annually thereafter, if the polyp size does not increase. For polyps sized 5 mm or less, a follow-up ultrasound of the gallbladder is not recommended.

In patients with primary sclerosing cholangitis, the incidence of intraepithelial neoplasia is high and the malignant potential of gallbladder polyps warrants regular screening for gallbladder cancer [79,91,92,93].

The American Association for the Study of Liver Diseases recommends a follow-up ultrasound every 6 months for gallbladder polyps 8 mm or less in patients with primary sclerosing cholangitis. For polyps more than 8 mm, the decision between cholecystectomy and follow-up is based on patient factors (baseline liver function, the risk of perioperative hepatic decompensation, and hepatobiliary infection) [94]. The American College of Gastroenterology recommends cholecystectomy for polyps more than 8 mm because of the increased risk of being or becoming malignant. Surveillance of the gallbladder for cancer on at least an annual basis with ultrasound in patients with primary sclerosing cholangitis is also recommended [95].

#### 2.1.2. Serum Markers

In a retrospective study, carcinoembryonic antigen (CEA) and carbohydrate antigen 19-9 (Ca19-9) failed to detect the presence of gallbladder cancer, particularly in its early stages [96]. The potential use of circulating tumor DNA, circulating microRNA, and circulating tumor cells in diagnosing gallbladder cancer is also promising. Although there have been noteworthy studies, more research is needed before liquid biopsies can be integrated into standard clinical practice [97,98,99]. Kumari et al. evaluated the diagnostic value of the level of circulating serum-free DNA (cfDNA) in the GBC. They compared the levels of cfDNA in 34 cases of GBC, 22 cases of cholecystitis, and 17 healthy controls using quantitative PCR (qPCR). Cancer patients had substantially higher cfDNA levels than cholecystitis patients and the control group [100]. In addition, a cfDNA cutoff value of >218.55 ng/mL distinguished cancer patients from healthy controls with 100 percent sensitivity (95% confidence interval (CI): 89.6–100; *p* 0.001) and 100 percent specificity (95% CI: 80.3–100; *p* 0.001). Shen et al. discovered a correlation between the molecular characteristics detected in BTC patient bile and tissue samples. Using targeted deep sequencing to compare biliary cfDNA and tumor DNA for single nucleotide variation (SNV)/insertion and deletion (Indel) and copy number variation (CNV), a high level of sensitivity (94.7% and 75%, respectively) and specificity (99.9% and 98.8%, respectively) was observed [101]. Using next-generation sequencing (NGS), Kinugasa et al. analyzed mutations in DNA extracted from bile and tumor tissue of 30 patients with GBC. The rate of mutation concordance between GBC tissue DNA and cfDNA was discovered to be 85.7%. The mutation frequencies detected in cfDNA were roughly half of those found in DNA extracted from tumor tissue [98].

ALU247 distinguished GBC (n = 60) from controls (n = 36) with a sensitivity, specificity, and diagnostic accuracy of 80%, 86.1%, and 82.2%, respectively, in a study evaluating long DNA fragments in serum samples [102].

Cancer antigen CA 242 is unaffected by inflammatory conditions. This marker demonstrated a remarkable 98.7% specificity for GBC diagnosis. The combination of CA 19-9, CA 125, and CA 242 increased diagnostic specificity but not sensitivity, reaching 100 percent specificity with a 100 percent positive predictive value (PPV) [103]. Prior to this study, Rana et al. reported that CA 242 has high specificity and PPV for the diagnosis of GBC, in addition to a high discriminatory potential for differentiating malignant from benign biliary disease [104]. CA 242 demonstrated superior diagnostic performance compared to CEA and CA 19-9, which had sensitivity and specificity values of 64% and 83%, respectively [104]. In spite of this, the diagnostic accuracy of these markers must be validated in a larger patient population.

Currently, there are no established screening recommendations for gallbladder cancer for use in the standard-risk population.

### 2.2. Cholangiocarcinoma (Intrahepatic, Perihilar, and Extrahepatic)

Cholangiocarcinomas (CCAs) are divided into three subtypes based on their anatomic location: intrahepatic (iCCA), perihilar (pCCA), and distal (dCCA) [105]. The epidemiology of cholangiocarcinomas has significant geographic variations. Infection with certain trematodes (Clonorchis sinensis and Opisthorchis viverrini), commonly referred to as flukes, is one of the leading causes of CCA in certain regions. In Southeast Asia, where fluke infection is endemic, the prevalence of CCA is notably high [106]. The attachment of liver flukes to the biliary wall results in ulceration and precancerous lesions [107]. Although there is evidence that certain risk factors may contribute to the disease in some patients, the majority of individuals with CCA in the Western world do not have any identifiable predisposing factors.

The most well-known risk factor for CCA is primary sclerosing cholangitis [108]. Caroli disease and choledochal cysts are two of the known risk factors for all three subtypes of CCA [109]. Similarly to gallbladder cancer, risk factors for cholangiocarcinomas are associated with the prevalence of chronic inflammation, but unlike gallbladder cancer, cholelithiasis is not associated with CCA [110]. Hepatitis B, non-alcoholic fatty liver disease, and cirrhosis are correlated more strongly with iCCA, whereas choledocholithiasis is correlated more strongly with pCCA and dCCA [109]. CCAs are typically discovered at an advanced stage and mortality is high; early detection is critical for providing treatment and reducing mortality. As with gallbladder cancer, it is critical to examine the known risk factors and identify the group to screen.

For fluke-related CCA in endemic areas, consuming raw, undercooked, fermented, or dried freshwater fish is the main risk [111]. The screening, diagnosis, and treatment of fluke infection should be addressed through preventive measures. Present or previous liver fluke infection can be detected via fecal, blood, and urine examinations for the presence of eggs, fluke antigens, antibodies, and nucleic acids [112,113,114]. Praziquantel, an anthelmintic medication given orally, is used to treat and cure infection [115]. Considering the high frequency of fluke infection and cholangiocarcinoma in specific geographical locations, serological tests for fluke infection and abdominal ultrasonography or other radiological imaging for cholangiocarcinoma screening have been performed [114,116,117,118]. Even while praziquantel effectively cures the infection, periductal fibrosis is rarely resolved, and imaging can show periductal fibrosis, which can lead to CCA [116,119]. The Cholangiocarcinoma Screening and Care Program (CASCAP) is a prospective cohort study for CCA screening and care at Khon Kaen University in Thailand to conduct community-based ultrasound screening programs for the early diagnosis of CCA [117,120]. For the screening cohort, an ultrasound examination is carried out regularly at least annually to determine whether there is a current bile duct or liver pathology. Individuals who were suspected of having CCA underwent confirmatory MRCP or computerized tomography (CT) scanning. It represents the most detailed and comprehensive study optimization of screening methods for the early diagnosis of CCA and the cohort results are going to guide screening measures for fluke-related CCA.

Primary sclerosing cholangitis (PSC) is a premalignant biliary tract disease that carries a significant risk of developing cholangiocarcinoma, often of the perihilar type [121]. However, there is no standardized surveillance measure for individuals with PSC. This is partly due to the challenging diagnosis of cholangiocarcinoma in PSC, as inflammation-related dominant biliary strictures can mimic the disease, and cytological techniques lack sensitivity, requiring invasive endoscopic procedures [112,121,122]. Annual magnetic resonance cholangiopancreatography (MRCP) surveillance of asymptomatic PSC patients may reduce mortality, with MRI being superior to ultrasound in detecting early-stage cholangiocarcinoma in PSC patients [112,123]. In a retrospective study which included all Mayo Clinic patients with a diagnosis of PSC and high serum CA 19-9 levels, it was found that more than one third of the patients did not have CCA [124]. Ultrasound has high specificity (94%) but low sensitivity (57%) for CCA in patients with primary sclerosing cholangitis compared to MRI/MRCP, with sensitivity of 89% and specificity of 75% [125]. The contributions of annual clinical follow-ups, liver function tests, contrast-enhanced MRI/MRCP, and carbohydrate antigen (CA) 19-9 in patients with PSC were investigated in a recent study [126]. Despite surveillance, only 2% of patients were diagnosed with cholangiocarcinoma, and their prognosis remained dismal. This strategy of surveillance failed to detect cancer early enough to promote long-term survival. Contrary to this study, it has been shown that screening patients with PSC, similar to the previous study, contributes to early diagnosis and survival [127,128]. The apparent survival advantage linked with yearly imaging surveillance may be attributable to lead-time bias, and the potential benefit of surveillance is yet-known. The European Association for the Study of the Liver recommends surveillance with ultrasound and/or MRI/MRCP for CCA at least yearly and every 6 months in the presence of cirrhosis. CA 19-9 is not recommended for surveillance due to its insufficient precision [129]. The American Association for the Study of Liver Diseases recommends annual CCA surveillance with abdominal imaging, preferably MRI/MRCP with or without serum CA 199, and intraductal tissue sampling for cytology and FISH during endoscopic retrograde cholangiopancreatography (ERCP) for relevant strictures [94]. The American College of Gastroenterology suggests screening for CCA with regular cross-sectional imaging with ultrasound or MR and serial CA 19-9 testing every six to twelve months. The guideline also recommends ERCP with cytology, biopsies, and FISH for PSC with an imaging-detected dominant stricture to rule out CCA. Both the American Association for the Study of Liver Diseases and the American College of Gastroenterology have stated that the predictive value of CA 19-9 is limited [94,95,124,130,131]. A cfDNA analysis of cholangiocarcinoma patients and healthy sex- and age-matched controls revealed differentially methylated regions (DMRs) in four genes (HOXA1, PRKCB, CYP26C1, and PTGDR) in CCA patients [132]. The panel showed specificity of 93% and sensitivity of 83% for the detection of cholangiocarcinoma; interestingly, the DMR ctDNA panel detected that 32 (80%) of the 40 CCAs were deemed eligible for surgical resection or transplantation and 15 (60%) of the 40 CCAs were deemed not eligible for surgical resection or transplantation. The possible uses of liquid biopsies in the diagnosis of cholangiocarcinoma, as in gallbladder cancer, are also promising, but more research is needed before they can be implemented into routine clinical practice [97,133].

## 3. Conclusions

While new treatment options have emerged for both HCC and BTC, advanced disease remains uniformly fatal. Therefore, early diagnosis is crucial for curing a significant portion of patients with early-stage disease. Since HCC often develops in the context of chronic liver disease, surveillance using imaging in patients with chronic liver disease is a reasonable approach. Currently, USG is the recommended and most commonly used modality due to its acceptable cost and wider availability. However, the limited sensitivity and specificity of USG can be improved by incorporating blood-based biomarkers. AFP has been used as a biomarker for many years, but it is not sufficient for screening purposes alone. Combining AFP with other markers has shown promising results. ctDNA, cfRNA, and extracellular vesicles, which contain circulating nucleic acids, have the potential to serve as biomarkers for HCC. Adding imaging and protein biomarkers to liquid biopsy biomarkers could improve sensitivity and specificity.

The early diagnosis of BTC is more challenging, as high-risk patient groups are less well defined, and imaging and blood-based biomarkers are less sensitive for BTC. Surveillance using USG is recommended for selected high-risk subgroups, including those with gallbladder polyps, porcelain gallbladder, and primary sclerosing cholangitis, and cholecystectomy may reduce the risk of gallbladder cancer if positive findings are detected. MRI/MRCP (magnetic resonance imaging/magnetic resonance cholangiopancreatography) has higher sensitivity and accuracy in diagnosing BTC and can be employed in selected patients at higher risk of BTC. The potential applications of ctDNA, circulating microRNA, and circulating tumor cells in the early diagnosis of BTC are active areas of research, and further studies are needed to demonstrate their clinical efficacy before they can be routinely used in clinical practice.
